# Knowledge in the Area of Prevention and Treatment of Pressure Injuries Among Nurses: Report from the Study

**DOI:** 10.3390/healthcare13010065

**Published:** 2025-01-02

**Authors:** Joanna Przybek-Mita, Dariusz Bazaliński, Ewelina Małek, Jan Kachaniuk, Dorota Kozieł, Maria Kózka, Maria Teresa Szewczyk

**Affiliations:** 1 Institute of Health Sciences, College of Medical Sciences, University of Rzeszów, 35-959 Rzeszów, Poland; 2 Postgraduate Nursing and Midwifery Education Centre, 35-083 Rzeszów, Poland; ewelin.malek@gmail.com; 3 Podkarpackie Specialist Oncology Centre, Father B. Markiewicz Specialist Hospital in Brzozów, 36-200 Brzozów, Poland; 4 Institute of Health Sciences, Faculty of Medicine, John Paul II Catholic University of Lublin, 20-708 Lublin, Poland; jan.kachaniuk@kul.pl; 5 Faculty of Health Sciences, Collegium Medicum, J. Kochanowski University, 25-317 Kielce, Poland; dkoziel@ujk.edu.pl; 6 Department of Clinical Nursing, Faculty of Health Sciences, Collegium Medicum, Jagiellonian University, 31-501 Krakow, Poland; maria.kozka@uj.edu.pl; 7 Department of Perioperative Nursing, Department of Surgical Nursing and Chronic Wound Care, Collegium Medicum in Bydgoszcz, Nicolaus Copernicus University in Torun, 85-821 Bydgoszcz, Poland; mszewczyk@cm.umk.pl

**Keywords:** pressure injury, knowledge, nurse, pressure injury prevention, pressure injury treatment

## Abstract

**Introduction**: Pressure injuries represent a significant issue in nursing care, with prevalence rates ranging from 5 to 27% among hospitalized patients and 3–32% in long-term care settings. Nurses’ knowledge of pressure injury prevention and treatment plays a crucial role in reducing their incidence. Objective: The aim of this study was to assess nurses’ knowledge of pressure injury prevention and treatment, taking into account their professional and postgraduate education, self-directed learning activities, and interest in wound care. **Materials and Methods**: This study included 500 nurses working in hospital wards and primary healthcare settings who participated in specialized nursing training programs. The Pieper–Zulkowski Pressure Ulcer Knowledge Test (PZ-PUKT), in its 2021 version and adapted into Polish was used to assess the nurses’ knowledge. The main study was conducted over five months, from March to July 2024. **Results**: This study revealed that nurses’ knowledge of pressure injury prevention and treatment was above average, with a mean score of 49.6 ± 5.2. **Conclusions**: The level of knowledge among the nurses assessed using the Polish version of the PZ-PUKT was above average. The PZ-PUKT test can be a useful tool for evaluating pressure injury knowledge among both nursing students and employed nurses and may serve as a means for identifying knowledge gaps in pressure injury prevention and treatment.

## 1. Introduction

Global epidemiological data indicate a high incidence of hard-to-heal wounds, particularly pressure injuries (PIs), vascular ulcers, and diabetes-related ulcers, especially in developing countries [1,2]. The presence of chronic, hard-to-heal, pressure-related wounds constitutes a significant burden not only for patients and their families but also for society and the healthcare system. Pressure injuries represent a significant issue in nursing care, with prevalence rates ranging from 5 to 27% among hospitalized patients and 3–32% in long-term care settings [2,3]. These wounds contribute to a diminished quality of life for patients and their families and can be a cause of premature death. Contributing factors include internal factors, which are linked to the patient’s health status, and external factors, which are influenced by the care provided. Risk factors for PIs in adult patients include physiological factors (advanced age, body mass index (BMI) < 18.5, malnutrition, incontinence, impaired mobility, poor perfusion, dehydration, and comorbid conditions such as diabetes), extrinsic factors that affect skin integrity (friction/shear, interface pressure and turning and repositioning) and mental status/neurological disorders (traumatic brain injury, dementia or other cognitive disorders). Despite research efforts into the etiopathogenesis of PIs across various patient populations, as well as the establishment of scientific evidence supporting effective prevention and treatment methods and the development of global guidelines, a declining trend in the incidence of this health and social issue has yet to be achieved. This situation is likely related to demographic changes that drive the global increase and aging of the human population.

Advanced age predisposes individuals to changes in the structure and function of the skin, and those with neurological dysfunction are particularly susceptible to skin damage caused by direct pressure and friction. The increased susceptibility stems from the aging processes of the skin itself, as well as systemic and chronic diseases. The issue may also be linked to delayed or unprofessional prevention [3]. Pressure injuries (PIs) are a common problem in hospitalized adults that adversely impact patient health and impose a substantial financial burden on the health system. In many countries there are also legal issues because PIs are considered as caused by inadequate care, and compensation for patients who developed PIs at hospitals has been as high as many millions of dollars [3,4].

One of the primary and essential steps in ensuring proper prevention, diagnosis, and treatment of pressure injuries by nurses is having adequate knowledge and professional preparation in this area [4,5]. Studies conducted in many countries have shown a deficiency in nurses’ knowledge regarding pressure injury prevention and treatment [6,7,8]. The knowledge level of health professionals can be assessed using standardized and culturally validated clinimetric tools, which allow for evaluating preparedness in wound prevention and care, identifying knowledge gaps, and implementing corrective educational actions [9]. Research conducted in Poland utilized the “Pressure Ulcer Knowledge Test” developed by Barbara Pieper and Karen Zulkowski (PZ-PUKT), 2021 version, as adapted into Polish by Joanna Przybek-Mita et al. [10,11,12].

## 2. Materials and Methods

### 2.1. Ethical Considerations

The study protocol was approved by the Bioethics Commission at the University of Rzeszów (Resolution No. 2018/01/07h, 11 January 2018), update 045/11/2024. The guidelines of the Helsinki Declaration were introduced in the course of the conducted study. Participants were informed about the purpose of this study and provided informed consent before starting this study, and they could withdraw at any point without giving a reason [13]. Permission to conduct the cultural adaptation process and psychometric studies of the PZ-PUKT was obtained from the authors, Barbara Pieper of the College of Nursing at Wayne State University de Detroit, Michigan, and Karen Zulkowski of the College of Nursing at Montana State University, Bozeman, Montana, in the United States.

### 2.2. Subject

Pressure ulcers are classified as pressure injuries (PIs) involving the skin and/or subcutaneous tissue and frequently occur over bony prominences due to a combination of the patient’s intrinsic factors and the effects of direct pressure and shear forces. In 2019, the National Pressure Injury Advisory Panel (NPIAP) and the Pan Pacific Pressure Injury Alliance (PPPIA) jointly established guidelines on pressure injuries. The Expert Panel [14] of the National Pressure Injury Advisory Panel (NPIAP) and the Pan Pacific Pressure Injury Alliance (PPPIA) developed a joint position on pressure injuries in 2019. The Polish guidelines from 2020, prepared by the expert team of the Polish Wound Management Association (in Polish, PTLR—Polskie Towarzystwo Leczenia Ran), are consistent with international guidelines [15,16]. Experts from global associations have highlighted an incidence rate of up to 72.5% in various countries, though the statistical data vary between countries and are presented in general terms [14]. Considering these expert guidelines and the epidemiology of pressure injuries, attention has been drawn to the need for assessing knowledge levels in this field. To date, no studies in Poland have been conducted using a standardized tool to assess knowledge on pressure injury prevention and treatment.

### 2.3. Research Method and Tool

In developing the methodological framework, the estimation method and diagnostic survey were applied. The research tool was a standardized knowledge test on pressure injuries developed at the University of South Australia, the Pieper–Zulkowski Pressure Ulcer Knowledge Test (PZ-PUKT), 2021 version [17,18]. Permission was obtained from the PZ-PUKT authors to prepare a Polish-language version. The PZ-PUKT test consisted of 72 items assessing knowledge across three subscales: prevention (31 items), pressure injury staging (21 items), and wound description (20 items). Each item had three response options (true, false, and don’t know), with participants receiving one point for each correct response and zero points for incorrect answers. Among the 72 statements, 42 were true, and 30 were false. The total score ranged from 0 to 72 points. The tool was user-friendly and effective in assessing knowledge of pressure injury prevention and treatment. The internal consistency coefficient for the Polish-adapted PZ-PUKT, as adapted by Przybek-Mita et al., was 0.776 for the overall test, 0.50 for the prevention subscale, 0.47 for the injury staging subscale, and 0.38 for the wound description subscale [12].

### 2.4. Course of This Study

The PZ-PUKT tool, preliminarily adapted to Polish conditions by Przybek-Mita et al. [12], was used in this study. Nurses participating in postgraduate clinical nursing education were eligible for this study. Out of a group of 1230 individuals, 612 voluntarily consented to the test assessment. This study was conducted during specialized nursing training sessions organized nationwide by the Postgraduate Education Center for Nurses and Midwives in Rzeszów (in Polish, OKPPiP—Ośrodek Kształcenia Podyplomowego Pielęgniarek i Położnych). Participation was voluntary and anonymous. Respondents received instructions for completing the questionnaire along with the PZ-PUKT form in Polish, and each participant had the option to withdraw from this study at any time. The allocated time for completing the tool was 60 min, though most participants completed the assessment in an average of 20 min. After submitting the questionnaire, participants were asked about any difficulties in understanding the adapted version of the PZ-PUKT. The pilot study was conducted in line with the methodological framework. For statistical analysis, 500 fully completed questionnaires were included, while 112 were excluded due to incomplete data exceeding 20%. All participants were actively engaged in specialized training organized by the Postgraduate Education Center for Nurses and Midwives in Rzeszów. Inclusion criteria were consent to participate, current employment as a nurse, interest in wound prevention and treatment, a minimum of 12 months of professional experience, and competencies in wound prevention and treatment. Exclusion criteria included lack of consent to participate and lack of current employment in a healthcare facility. The questionnaires were personally distributed to respondents, following an oral explanation of this study’s purpose. This study was conducted in 12 training groups between March and July 2024.

### 2.5. Statistical Analysis

Statistical analysis was performed using IBM SPSS v21. The Kolmogorov–Smirnov test was used to assess the normality of distributions, and Cronbach’s alpha coefficient was applied to evaluate the reliability of the scales. Since both the independent variables (age, self-assessment of knowledge, interest in wound care) and the PZ-PUKT scales did not follow a normal distribution, Spearman’s rho correlation coefficient and non-parametric significance tests for distribution differences (Mann–Whitney U test and Kruskal–Wallis test) were used to assess the relationships between variables.

## 3. Results

### 3.1. Characteristics of the Study Participants

The age of the participants ranged from 25 to 66 years, with an average age of 41.73 ± 10.31 years. The majority of respondents (77.2%) held higher education degrees, with 46.4% having completed a master’s degree and 32.8% a bachelor’s degree. All participants were certified in pressure injury prevention and independently qualified to select appropriate treatment methods for pressure injuries in Poland. The nurses surveyed were employed in various settings, with the majority (68.6%) working in hospital departments (46.6% in surgical units and 22% in non-surgical units). Detailed data on the study group are illustrated in Table 1.

### 3.2. Self-Assessment of Knowledge on the Prevention and Treatment of Pressure Injuries

The first part of the tool consisted of questions related to self-assessment of knowledge about wound treatment, where 0 indicated no knowledge, and 10 indicated a high level of knowledge. The largest group of respondents, 99.2%, rated their knowledge in this area as above average or high, with 34.8% rating it as below average or low and 26% as average (Table 2). Regarding interest in the topic of pressure injury prevention and treatment, 68.8% of responses fell in the above-average and high categories, 15.6% at the average level, and 15.6% below average and low (Table 3). Descriptive statistics of the responses are presented in Table 4.

### 3.3. Assessment of Knowledge Using the PZ-PUKT Test

The PZ-PUKT questionnaire was used to assess knowledge in the area of pressure injury prevention and treatment. The study group scored between 25 and 63 points on the test (mean 49.6 ± 5.2). Correct answers were provided by 68.9% of the respondents throughout the entire pressure injury knowledge test. Similar results were obtained for each of the three subscales: prevention, stage of pressure injury, and wound description (Table 5).

Analyzing the detailed data from all 72 questions included in the three categories of the PZ-PUKT questionnaire, it was noted that most of the component questions did not pose difficulties for the respondents, and the answers in the true and false categories were accurately assigned. A detailed analysis of the questions revealed that the highest number of incorrect responses was given in the domain concerning wound assessment, while the domain dedicated to prevention yielded the best results in terms of knowledge. The distribution of responses in the prevention domain for individual questions ranged from 4% to 97%. The affirmative statements were the best understood and confirmed the respondents’ existing knowledge. Among the 18 questions posed, the vast majority of respondents selected the correct answers, with scores ranging from 71% to 97%. The questions regarding pressure injury prevention, where false statements were presented, proved to be slightly more challenging. The overall performance of the group was in the range of from 4% to 91%. Out of 13 questions, as many as five were either poorly understood or indicated inadequate knowledge in this area. The question “Donut devices/ring cushions help to prevent pressure ulcers/injuries” posed the greatest difficulty, with only 4% of respondents providing the correct answer. This study also indicated an underestimation of staff education in the area of pressure injury prevention (78%), as only 22% of nurses affirmed that “education of the staff alone can reduce the incidence of pressure ulcers.” Another challenging question was formulated as “Persons, who are immobile and can be taught, should shift their weight every 30 min while sitting in a chair”, to which only 28% of respondents provided the correct answer. Additionally, the question “Massage of bony prominences is essential for quality skin care” posed challenges for a significant portion of respondents, as 62% gave an incorrect response. The final question that recorded more than half of the incorrect answers was “Alkaline soap products should be used to cleanse soiled skin”, which was answered correctly by only 46% of nurses. In conclusion, out of 31 statements in the prevention domain of the PZ-PUKT test, only five questions proved difficult for the respondents to answer correctly.

In the section of the test assessing the stage of the wound, 21 questions were posed, of which 9 were affirmative questions, and 12 required a negative response. For the affirmative questions, respondents achieved a score ranging from 62% to 94% for correct answers. Conversely, the section of the questionnaire formulated in the form of negations posed difficulties for five questions, where more than half of the respondents answered incorrectly. The highest rate of incorrect answers, at 74%, was noted for the question “Non-blanchable erythema anywhere in the body is a stage 1 pressure ulcer/injury”, followed by the question “Pressure ulcers/injuries progress in a linear fashion from stage 1 to 2 to 3 to 4”, for which only 28% knew the correct answer. Significant difficulties were also observed with the questions “A stage 3 pressure ulcer/injury is a partial thickness skin loss involving the epidermis and/or dermis”—with 68% incorrect answers; “Stage 4 pressure ulcers/injuries always have undermining”—with 65% incorrect answers; and “Skin tears are classified as stage 2 pressure ulcers/injuries”—with 60% incorrect answers provided. The remaining questions did not pose significant difficulties, achieving correct answer rates ranging from 50% to 89%.

The third section of the PZ-PUKT test contained 30 questions focused on wound description, including 8 questions phrased positively and 12 questions where the correct answer required contradicting the stated premise. Responses to the affirmative statements (“true”) did not pose difficulties for the respondents, with correct answers ranging from 70% to 94%. Greater difficulty was observed in questions where the premise needed to be negated, with correct responses ranging from 23% to 90% depending on the question. The highest rates of incorrect answers were recorded for the following questions: “Wounds that become chronic are frequently stalled in the proliferative phase of healing”, 72% incorrect answers; “Pressure ulcers/injuries should not be cleansed with drinking water”, 69% incorrect selections; and “ABD pads may be used to protect the skin”, 66% incorrect answers. A substantial portion of respondents also struggled to correctly answer questions such as “Hydrocolloid dressings should be used on stage 2 infected ulcer/injury”, 62% incorrect answers, and “Wound biofilm is associated with decreased wound drainage”, 50% incorrect answers and 2% “don’t know” responses. In the remaining questions, over half of the responses were correct, indicating that respondents generally had no major issues distinguishing between true and false statements. The entire tool with response categories is presented in Table 6.

### 3.4. Self-Assessment of Knowledge and Interest in Wound Treatment Versus PZ-PUKT Assessment

For self-assessed wound care knowledge and interest in wound treatment, the relationship between these variables and PZ-PUKT scales was examined using Spearman’s rho correlation coefficient (0.187) and the Kruskal–Wallis test (for grouped variables). Both variables are significantly associated with PZ-PUKT scales; both Spearman’s rho correlations and Kruskal–Wallis tests indicate statistically significant differences (*p* < 0.0001) in the distribution of PZ-PUKT scores across categories defined by self-assessed wound care knowledge and interest levels (see Table 7 and Table 8). Statistically significant correlations (*p* < 0.05) for wound interest were found specifically within the Wound subscale. However, the values of Spearman’s rho, including those that were statistically significant, were low (*p* < 0.01). The average percentage of correct answers on PZ-PUKT scales increases with higher self-assessed knowledge of wound care. For interest in wound care, the highest average percentage of correct answers on PZ-PUKT scales was observed among participants rating their interest at a medium level—6–7 on a scale of 0–10.

## 4. Discussion

Global trends in nursing practice emphasize the need to maintain professional autonomy and possess high professional competencies, which, in turn, requires a strong focus on an appropriate level of nursing knowledge and skills [10,19]. This is particularly important in the prevention and treatment of pressure injuries (PIs) and other hard-to-heal wounds, as epidemiological data indicate increasing trends associated with the aging population, lack of mobility, malnutrition in elderly individuals, and the presence of comorbidities that impair the functioning of the body and wound healing [3,14]. Research conducted in developed countries suggests that chronic wounds occur in 1–2% of the population [20], while data from the systematic review by Martini et al. [21] show a prevalence of mixed etiology wounds at 2.21 per 1000 individuals. The most common wounds include venous leg ulcers (VLUs), pressure injuries (PIs), and diabetic foot ulcers (DFUs) in individuals over 60 years of age. Data indicate that pressure injuries rank third in frequency, just after diabetic foot disease and tropical ulcerations of the lower legs [19]. A certain percentage of wounds may not heal completely within a year or longer, which represents a significant burden on healthcare systems [22].

In the conducted study, the primary focus was on assessing the knowledge of practicing nurses regarding the prevention and treatment of pressure injuries, as appropriate health interventions applied in a timely manner should form the foundation of every nurse’s practice, regardless of the setting in which healthcare services are provided. In Poland, nurses are authorized to prevent and treat wounds either upon a doctor’s order or independently, provided they possess the competencies defined in the Ministry of Health regulation from 2017 [23]. The provisions of this legal act specify the level of professional education necessary for independent intervention in wound treatment, which is defined based on the completion of a specialized course in wound treatment, a qualifying course, holding a specialist title in nursing if the course or specialization program included educational content in this area, or holding a master’s degree in nursing. Completion of a second-degree program in nursing or appropriate forms of postgraduate education should equip nurses with the requisite level of knowledge and practical skills, which require continuous updating throughout their professional lives.

Assessing and evaluating nurses’ knowledge about the prevention and treatment of pressure injuries using standardized tools allows for the identification of knowledge deficits in this area and may serve as a basis for corrective actions in both education and nursing practice. Most tissue injuries of pressure ulcer etiology (95%) are considered preventable if appropriate remedial measures are taken [15,16]. The appearance of pressure injuries is a source of pain, reduces the quality of life, and affects the longevity of the patient, as well as prolongs the patient’s stay in a medical facility, which significantly impacts healthcare costs. To reduce the risk of pressure injuries, effective and safe actions should be taken as described in the 2019 guidelines and recommendations by the European Pressure Ulcer Advisory Panel (EPUAP) and the National Pressure Injury Advisory Panel (NPIAP) [3,14]. Despite the individual needs of each patient, varying levels of cooperation in the prevention and treatment of pressure injuries due to health status and comorbidities, as well as the location of healthcare services, the implementation of guidelines into practice should always be considered. The recommendations in the EPUAP/NPIAP Guidelines focus on analyzing risk factors and assessing their impact on the development of pressure injuries, evaluating skin quality, principles of preventive skin care, assessing the nutritional status of the patient, changing body positions, and early mobilization of the patient, as well as analyzing the risk of pressure injury associated with heel pressure [14].

The guidelines also include recommendations regarding the selection of beds, mattresses, and other support surfaces, as well as the prevention of injuries caused by medical devices used. They provide information on the classification of pressure injuries, assessment of ulcer stage and monitoring of the healing process, assessment and treatment of pain associated with pressure injuries, the occurrence of infection and/or biofilm, as well as practices for cleansing the ulcer during treatment, optimal dressing selection, and the use of electrical stimulation or negative pressure in the treatment process. Furthermore, they define indications for surgical treatment of pressure injuries. The EPUAP/NPIAP guidelines serve as a foundational document implemented in practice as evidence-based medicine (EBM), but they also highlight issues that require further in-depth research in this area. In Poland, recommendations/guidelines regarding the prevention and treatment of pressure injuries were formulated by experts from the Polish Wound Management Association (in Polish, PTLR—Polskie Towarzystwo Leczenia Ran) in 2020 [15,16]. They result from the adaptation of global research findings to the functioning conditions of the Polish healthcare system, taking into account economic, staffing, and legal possibilities. The recommendations formulated therein can serve as a valuable source of knowledge for nurses regarding the prevention and treatment of pressure injuries. Nurses are obliged to undertake multifaceted actions in effective prevention and, in the event of pressure injuries, to initiate effective treatment, provided they possess appropriate, documented qualifications. The implementation of proper preventive and therapeutic measures requires up-to-date knowledge and skills in wound treatment. Both the EPUAP/NPIAP guidelines and the Polish Wound Management Association recommendations organize knowledge, provide support, and serve as a kind of “roadmap” for action [10,15,19].

This is extremely important and can be helpful in standardizing nursing services in the prevention and treatment of pressure injuries. It requires an understanding of the processes occurring in healthy tissues and, in hard-to-heal wounds, clinical assessment of the patient and the pressure area, proper and conscious preventive and therapeutic measures, knowledge of the beneficial effects and burdens as well as side effects of various pressure injury treatment therapies, and the ability to utilize best practices. However, research indicates that there are certain difficulties in implementing the accepted recommendations regarding the prevention and treatment of pressure injuries due to the attitudes of interdisciplinary team members. Effective implementation requires strategies that promote better dissemination of recommendations in clinical practice [3,19,24]. The strategy used in most countries around the world for preventing and treating pressure injuries encompasses three main areas: risk assessment for pressure injury development, conducting multi-level prevention, and providing multifaceted treatment when a pressure injury occurs [15,16,25]. In this context, the role of nursing staff is invaluable and important, as they provide direct care for patients both in hospital and home care settings. Given the above, reliable knowledge and skills of nurses are crucial for conducting effective prevention and treatment of pressure injuries [26,27,28]. Unfortunately, research conducted in various parts of the world regarding nursing practice indicates certain knowledge deficits in the area of pressure injury prevention [4,9,11,29,30].

In this study, the level of knowledge was assessed using the Pressure Ulcer Knowledge Test (PZ-PUKT) developed by Piepe–Zulkowski in its 2021 version, adapted to Polish conditions by Przybek–Mita and colleagues, highlighting thematic areas that require educational reinforcement. The analysis of the entire test and its individual subscales showed a level of knowledge above average, indicating that the correctness of the given answers exceeded 60%. A tendency to avoid the neutral answer “I don’t know” was observed, indicating greater confidence in their knowledge or existing beliefs regarding the effectiveness of the respondents’ actions in this study. Individuals indicating an average or above-average interest in wound treatment achieved higher scores on the PZ-PUKT assessment (*p* < 0.001). When assessing the knowledge of the surveyed nurses, it should be noted that the obtained results are consistent with those of other studies conducted in China [31], Iran [18], Brazil [32], and Portugal [33].

When comparing the knowledge results presented in this study with the Polish adaptation of the PZ-PUKT test conducted by Przybek-Mita et al. [12], which aimed to assess test consistency and question construction quality, three questions were identified as having extreme values in the difficulty index (DIFF) and discrimination index (DISCR). These values may suggest either poor question construction, making it difficult to understand the text or an insufficient knowledge level among respondents. The low index applied to the following questions: “Non-blanchable erythema anywhere in the body is a stage 1 pressure ulcer/injury” (question 12), “Donut devices/ring cushions help to prevent pressure ulcers/injuries” (question 26), and “Staff education alone may reduce the incidence of pressure ulcers/injuries” (question 34). These results may necessitate revising the questions and/or developing a shortened version of the test as, despite its simple response structure, the tool is lengthy and requires full attention and several dozen minutes to complete. However, the study authors, despite identifying questions with low discrimination indices, concluded that the PZ-PUKT questionnaire meets basic quality and consistency standards for questions and that the tool can be used to assess nurses’ knowledge in the prevention and treatment of pressure injuries, particularly among nursing students and participants in postgraduate training programs that authorize independent wound treatment. Knowledge tests can help non-medical health professionals and employers in planning activities related to education and increasing educational resources for those who most need to expand their knowledge. The use of shortened versions of the PZ-PUKT can be applied in studies of caregivers and assistants of elderly individuals to raise awareness of pressure injury prevention in this group.

## 5. Conclusions

The level of knowledge among the surveyed nurses, assessed with the Polish version of the PZ-PUKT test, as well as their interest in wound care, is above average. Using a standardized test within a group of health professionals qualified in wound prevention and treatment enabled an evaluation of knowledge about pressure injuries, highlighting strengths and weaknesses in areas related to pressure injury prevention and treatment. Further research on knowledge levels is recommended, particularly during higher education and specialization courses. The results should be evaluated and collected from academic centers nationwide to systematize and evaluate this area of knowledge for future pressure injury prevention and treatment recommendations.

## Figures and Tables

**Table 1:** Sociodemographic characteristics of participants (n = 500).

	n	%
Age		
up to 29	84	16.8%
30–39	131	26.2%
40–49	150	30.0%
50–59	118	23.6%
60+	17	3.4%
Gender		
Female	478	95.6%
Male	22	4.4%
Professional education		
Secondary	104	20.8%
Bachelor’s degree	164	32.8%
Master’s degree	232	46.4%
Additional postgraduate qualifications		
Specialization	81	16.2%
Courses for wound treatment	46	9.2%
Practice location		
Private practice	20	4.0%
Primary health care	62	12.4%
Surgical department (hospital)	218	43.6%
Non-surgical department (hospital)	110	22.0%
Long-term care	36	7.6%
Home hospice	13	2.6%
Specialized outpatient clinic (aos)	10	2.0%
Other	31	6.2%
Total	500	100%

**Table 2:** Self-assessment of current knowledge of wound treatment (pressure injury).

	n	%
0—No Knowledge	0	0.0%
1	8	1.6%
2	24	4.8%
3	58	11.6%
4	84	16.8%
5	130	26.0%
6	78	15.6%
7	53	10.6%
8	48	9.6%
9	10	2.0%
10—High Level of Knowledge	7	1.4%
Total	500	100.0%

**Table 3:** Interest in the topic of pressure injury care and treatment.

	n	%
0—No Interest	0	0.6%
1	7	1.4%
2	9	1.8%
3	27	5.4%
4	35	7.0%
5	78	15.6%
6	60	12.0%
7	62	12.4%
8	92	18.4%
9	49	9.8%
10—High Level of Interest	81	16.2%
Total	500	100.0%

**Table 4:** Descriptive statistics of self-assessment of knowledge and interest variables.

	Mean	Median	Minimum	Maximum	Standard Deviation	Valid n
At what level do you assess your current knowledge about PI treatment?	5.2	5	0	10	1.9	500
At what level do you assess your interest in PI treatment as an advanced professional practice?	6.8	7	0	10	2.3	500
				Kolmogorov–Smirnov ^a^		
				Statistic	df	Sig.
At what level do you assess your current knowledge about PI treatment?				0.149	500	0.000
At what level do you assess your interest in PI treatment as an advanced professional practice?				0.139	500	0.000

^a^–Lilliefors Significance Correction.

**Table 5:** Distribution of PZ-PUKT Subscales (n = 500).

	Number of Indicators	Mean	Median	Minimum	Maximum	Standard Deviation	% of Correct Answers	n
PZ-PUKT Scale	72	49.6	50	25	63	5.2	68.9%	500
Prevention	31	22.7	23	10	29	2.5	73.3%	500
Stage	21	13.3	13	6	19	2.4	63.5%	500
Wound	20	13.5	14	5	19	2.1	67.7%	500

**Table 6:** Distribution of responses in PZ-PUKT test questions (n = 500).

NR	Correct Answer	Subscale	True		False		Don’t Know	
			n	%	n	%	n	%
1	True	Wound	392	78.4%	95	19.0%	13	2.6%
2	False	Wound	46	9.2%	448	89.6%	6	1.2%
3	False	Wound	71	14.2%	418	83.6%	11	2.2%
4	False	Prevention	248	49.6%	230	46.0%	22	4.4%
5	True	Prevention	432	86.4%	63	12.6%	5	1.0%
6	False	Stage	339	67.8%	158	31.6%	3	0.6%
7	False	Wound	212	42.4%	277	55.4%	11	2.2%
8	True	Prevention	432	86.4%	66	13.2%	2	0.4%
9	True	Prevention	403	80.6%	92	18.4%	5	1.0%
10	False	Stage	348	69.6%	141	28.2%	11	2.2%
11	False	Wound	161	32.2%	326	65.2%	13	2.6%
12	False	Stage	369	73.8%	126	25.2%	5	1.0%
13	False	Prevention	153	30.6%	342	68.4%	5	1.0%
14	False	Stage	197	39.4%	300	60.0%	3	0.6%
15	True	Prevention	469	93.8%	31	6.2%	0	0.0%
16	True	Prevention	408	81.6%	89	17.8%	3	0.6%
17	False	Prevention	44	8.8%	453	90.6%	3	0.6%
18	True	Wound	451	90.2%	46	9.2%	3	0.6%
19	True	Prevention	417	83.4%	76	15.2%	7	1.4%
20	True	Prevention	411	82.2%	66	13.2%	23	4.6%
21	False	Stage	220	44.0%	269	53.8%	11	2.2%
22	True	Stage	444	88.8%	54	10.8%	2	0.4%
23	True	Prevention	455	91.0%	44	8.8%	1	0.2%
24	True	Prevention	429	85.8%	65	13.0%	6	1.2%
25	False	Stage	143	28.6%	351	70.2%	6	1.2%
26	False	Prevention	475	95.0%	21	4.2%	4	0.8%
27	True	Prevention	414	82.8%	80	16.0%	6	1.2%
28	False	Wound	330	66.0%	158	31.6%	12	2.4%
29	True	Prevention	486	97.2%	12	2.4%	2	0.4%
30	True	Wound	404	80.8%	91	18.2%	5	1.0%
31	True	Prevention	452	90.4%	42	8.4%	6	1.2%
32	True	Stage	431	86.2%	60	12.0%	9	1.8%
33	False	Stage	58	11.6%	441	88.2%	1	0.2%
34	False	Prevention	388	77.6%	111	22.2%	1	0.2%
35	True	Stage	362	72.4%	136	27.2%	2	0.4%
36	False	Prevention	173	34.6%	321	64.2%	6	1.2%
37	False	Stage	226	45.2%	266	53.2%	8	1.6%
38	True	Wound	349	69.8%	141	28.2%	10	2.0%
39	False	Wound	251	50.2%	242	48.4%	7	1.4%
40	True	Stage	310	62.0%	185	37.0%	5	1.0%
41	False	Wound	362	72.4%	116	23.2%	22	4.4%
42	False	Wound	201	40.2%	296	59.2%	3	0.6%
43	True	Stage	325	65.0%	172	34.4%	3	0.6%
44	False	Prevention	311	62.2%	184	36.8%	5	1.0%
45	True	Prevention	469	93.8%	30	6.0%	1	0.2%
46	True	Prevention	414	82.8%	80	16.0%	6	1.2%
47	True	Prevention	451	90.2%	45	9.0%	4	0.8%
48	True	Wound	409	81.8%	88	17.6%	3	0.6%
49	False	Stage	232	46.4%	248	49.6%	20	4.0%
50	True	Stage	468	93.6%	30	6.0%	2	0.4%
51	False	Prevention	350	70.0%	138	27.6%	12	2.4%
52	True	Stage	375	75.0%	120	24.0%	5	1.0%
53	True	Stage	422	84.4%	73	14.6%	5	1.0%
54	False	Prevention	228	45.6%	265	53.0%	7	1.4%
55	False	Prevention	182	36.4%	297	59.4%	21	4.2%
56	False	Prevention	99	19.8%	394	78.8%	7	1.4%
57	False	Prevention	110	22.0%	382	76.4%	8	1.6%
58	False	Prevention	93	18.6%	406	81.2%	1	0.2%
59	True	Prevention	474	94.8%	26	5.2%	0	0.0%
60	True	Wound	472	94.4%	27	5.4%	1	0.2%
61	False	Wound	93	18.6%	404	80.8%	3	0.6%
62	True	Prevention	452	90.4%	46	9.2%	2	0.4%
63	False	Stage	301	60.2%	187	37.4%	12	2.4%
64	True	Stage	427	85.4%	70	14.0%	3	0.6%
65	False	Wound	310	62.0%	180	36.0%	10	2.0%
66	True	Prevention	356	71.2%	141	28.2%	3	0.6%
67	False	Wound	346	69.2%	146	29.2%	8	1.6%
68	True	Wound	408	81.6%	79	15.8%	13	2.6%
69	False	Stage	53	10.6%	445	89.0%	2	0.4%
70	False	Wound	60	12.0%	434	86.8%	6	1.2%
71	True	Wound	437	87.4%	60	12.0%	3	0.6%
72	False	Stage	324	64.8%	170	34.0%	6	1.2%

**Table 7:** Average percentage of correct answers on PZ-PUKT scales by self-assessment categories of wound care knowledge.

Self-Assessment	PZ-PUKT Scale %		Prevention %		Stage %		Wound %	
	Mean	Standard Deviation	Mean	Standard Deviation	Mean	Standard Deviation	Mean	Standard Deviation
0–5	68.1	7.2	73.0	8.0	62.3	11.5	66.5	10.6
6–7	69.5	6.6	73.3	8.4	64.4	10.5	68.9	9.1
8–10	71.6	7.7	75.2	8.4	67.0	11.1	70.6	10.3
Total	68.9	7.2	73.3	8.2	63.5	11.3	67.7	10.3

**Table 8:** Average percentage of correct answers on the PZ-PUKT scales by interest in wound treatment.

Interest	PZ-PUKT Scale %		Prevention %		Stage %		Wound %	
	Mean	Standard Deviation	Mean	Standard Deviation	Mean	Standard Deviation	Mean	Standard Deviation
0–5	67.1	7.9	71.9	8.6	61.7	11.8	65.3	11.6
6–7	70.1	6.6	74.9	7.5	64.6	11.9	68.6	9.2
8–10	69.5	6.8	73.5	8.1	64.2	10.4	68.8	9.7
Total	68.9	7.2	73.3	8.2	63.5	11.3	67.7	10.3

## Data Availability

The original work presented in this study is included in the article. Further inquiries may be directed to the corresponding authors.
